# Supplementary material for: A fault-intrusion-tolerant system and deadline-aware algorithm for scheduling scientific workflow in the cloud
Source: PeerJ Comput Sci. 2021 Nov 2;7:e747. doi: 10.7717/peerj-cs.747 (PMC8576554; doi:10.7717/peerj-cs.747)
Supplement: Supplemental Information 6 [file peerj-cs-07-747-s006.zip › Myproject1.1/docs/org/cloudbus/cloudsim/power/class-use/PowerHost.html]

Uses of Class org.cloudbus.cloudsim.power.PowerHost


JavaScript is disabled on your browser.


- Overview
- Package
- Class
- Use
- Tree
- Deprecated
- Index
- Help

- Prev
- Next

- Frames
- No Frames

- All Classes

## Uses of Class org.cloudbus.cloudsim.power.PowerHost

- Packages that use PowerHost

  | Package | Description |
  | --- | --- |
  |  |  |
  | --- | --- |
  | org.cloudbus.cloudsim.examples.power |  |
  | org.cloudbus.cloudsim.power |  |
- - ### Uses of PowerHost in org.cloudbus.cloudsim.examples.power

    Fields in org.cloudbus.cloudsim.examples.power with type parameters of type PowerHost

    | Modifier and Type | Field and Description |
    | --- | --- |
    |  |  |
    | --- | --- |
    | `protected static java.util.List<PowerHost>` | RunnerAbstract.`hostList` The host list. |

    Methods in org.cloudbus.cloudsim.examples.power that return types with arguments of type PowerHost

    | Modifier and Type | Method and Description |
    | --- | --- |
    |  |  |
    | --- | --- |
    | `static java.util.List<PowerHost>` | Helper.`createHostList(int hostsNumber)` Creates the host list. |

    Method parameters in org.cloudbus.cloudsim.examples.power with type arguments of type PowerHost

    | Modifier and Type | Method and Description |
    | --- | --- |
    |  |  |
    | --- | --- |
    | `static Datacenter` | Helper.`createDatacenter(java.lang.String name, java.lang.Class<? extends Datacenter> datacenterClass, java.util.List<PowerHost> hostList, VmAllocationPolicy vmAllocationPolicy)` Creates the datacenter. |
  - ### Uses of PowerHost in org.cloudbus.cloudsim.power

    Subclasses of PowerHost in org.cloudbus.cloudsim.power

    | Modifier and Type | Class and Description |
    | --- | --- |
    |  |  |
    | --- | --- |
    | `class` | `PowerHostUtilizationHistory` The class of a host that stores its CPU utilization history. |

    Methods in org.cloudbus.cloudsim.power that return PowerHost

    | Modifier and Type | Method and Description |
    | --- | --- |
    |  |  |
    | --- | --- |
    | `PowerHost` | PowerVmAllocationPolicyAbstract.`findHostForVm(Vm vm)` Find host for vm. |
    | `PowerHost` | PowerVmAllocationPolicyMigrationAbstract.`findHostForVm(Vm vm)` Find host for vm. |
    | `PowerHost` | PowerVmAllocationPolicyMigrationAbstract.`findHostForVm(Vm vm, java.util.Set<? extends Host> excludedHosts)` Find host for vm. |
    | `protected PowerHost` | PowerVmAllocationPolicyMigrationAbstract.`getUnderUtilizedHost(java.util.Set<? extends Host> excludedHosts)` Gets the under utilized host. |

    Methods in org.cloudbus.cloudsim.power that return types with arguments of type PowerHost

    | Modifier and Type | Method and Description |
    | --- | --- |
    |  |  |
    | --- | --- |
    | `protected java.util.List<PowerHost>` | PowerVmAllocationPolicyMigrationAbstract.`extractHostListFromMigrationMap(java.util.List<java.util.Map<java.lang.String,java.lang.Object>> migrationMap)` Extract host list from migration map. |
    | `protected java.util.List<PowerHost>` | PowerVmAllocationPolicyMigrationAbstract.`getSwitchedOffHosts()` Gets the switched off host. |

    Methods in org.cloudbus.cloudsim.power with parameters of type PowerHost

    | Modifier and Type | Method and Description |
    | --- | --- |
    |  |  |
    | --- | --- |
    | `protected boolean` | PowerVmAllocationPolicyMigrationAbstract.`areAllVmsMigratingOutOrAnyVmMigratingIn(PowerHost host)` Checks whether all vms are in migration. |
    | `protected double` | PowerVmAllocationPolicyMigrationLocalRegression.`getMaximumVmMigrationTime(PowerHost host)` Gets the maximum vm migration time. |
    | `protected double` | PowerVmAllocationPolicyMigrationAbstract.`getMaxUtilizationAfterAllocation(PowerHost host, Vm vm)` Gets the power after allocation. |
    | `protected java.util.List<PowerVm>` | PowerVmSelectionPolicy.`getMigratableVms(PowerHost host)` Gets the migratable vms. |
    | `protected double` | PowerVmAllocationPolicyMigrationAbstract.`getPowerAfterAllocation(PowerHost host, Vm vm)` Gets the power after allocation. |
    | `protected double` | PowerVmAllocationPolicyMigrationAbstract.`getUtilizationOfCpuMips(PowerHost host)` Gets the utilization of the CPU in MIPS for the current potentially allocated VMs. |
    | `protected java.util.List<? extends Vm>` | PowerVmAllocationPolicyMigrationAbstract.`getVmsToMigrateFromUnderUtilizedHost(PowerHost host)` Gets the vms to migrate from under utilized host. |
    | `abstract Vm` | PowerVmSelectionPolicy.`getVmToMigrate(PowerHost host)` Gets the vms to migrate. |
    | `Vm` | PowerVmSelectionPolicyMaximumCorrelation.`getVmToMigrate(PowerHost host)` |
    | `Vm` | PowerVmSelectionPolicyMinimumMigrationTime.`getVmToMigrate(PowerHost host)` |
    | `Vm` | PowerVmSelectionPolicyMinimumUtilization.`getVmToMigrate(PowerHost host)` |
    | `Vm` | PowerVmSelectionPolicyRandomSelection.`getVmToMigrate(PowerHost host)` |
    | `protected abstract boolean` | PowerVmAllocationPolicyMigrationAbstract.`isHostOverUtilized(PowerHost host)` Checks if is host over utilized. |
    | `protected boolean` | PowerVmAllocationPolicyMigrationInterQuartileRange.`isHostOverUtilized(PowerHost host)` Checks if is host over utilized. |
    | `protected boolean` | PowerVmAllocationPolicyMigrationLocalRegression.`isHostOverUtilized(PowerHost host)` Checks if is host over utilized. |
    | `protected boolean` | PowerVmAllocationPolicyMigrationMedianAbsoluteDeviation.`isHostOverUtilized(PowerHost host)` Checks if is host over utilized. |
    | `protected boolean` | PowerVmAllocationPolicyMigrationStaticThreshold.`isHostOverUtilized(PowerHost host)` Checks if is host over utilized. |
    | `protected boolean` | PowerVmAllocationPolicyMigrationAbstract.`isHostOverUtilizedAfterAllocation(PowerHost host, Vm vm)` Checks if is host over utilized after allocation. |

- Overview
- Package
- Class
- Use
- Tree
- Deprecated
- Index
- Help

- Prev
- Next

- Frames
- No Frames

- All Classes
